# Does Amount of Pre-cue Encoding Modulate Selective List Method Directed Forgetting?

**DOI:** 10.3389/fpsyg.2020.01403

**Published:** 2020-07-31

**Authors:** Oliver Kliegl, Bernhard Pastötter, Karl-Heinz T. Bäuml

**Affiliations:** ^1^Department of Psychology, Regensburg University, Regensburg, Germany; ^2^Department of Psychology, University of Trier, Trier, Germany

**Keywords:** episodic memory, forgetting, directed forgetting, selectivity, list length

## Abstract

Prior work reported evidence that when people are presented with both a relatively short list of relevant information and a relatively short list of irrelevant information, a subsequent cue to forget the irrelevant list can induce successful selective directed forgetting of the irrelevant list without any forgetting of the relevant list. The goal of the present study is to determine whether this selectivity effect is restricted to short lists of information (six items per list), or if the effect generalizes to longer lists (12 items per list). In Experiment 1, we replicate the finding that selective directed forgetting can occur when short lists of relevant and irrelevant information are involved. Going beyond this replication, we show in Experiment 2 that such selectivity can arise both when shorter and when relatively long lists of items are used. The results are consistent with the view that selective directed forgetting can result from the action of a flexible inhibitory mechanism. They are less well in line with the view that selective cues to forget pre-cue information induce a change in participants' mental context.

## 1. Introduction

A necessary pre-requisite for targeted access to relevant memory content is constant updating of the memory system (Bjork, 1989). Research over the past six decades has shown that one way in which such updating can be achieved is to deliberately forget previously learned irrelevant information. Such intentional forgetting is required in a wide variety of everyday situations, such as when we learn that some study material is relevant for a later exam but other study material is not, and we want to forget the irrelevant information, or when some news we read on the internet turns out to be fake while other news appears to be credible, and we seek to forget the false information. In the laboratory, intentional forgetting has been demonstrated using the so-called list method directed forgetting (LMDF) task. In this task, participants are asked to study two lists of items and, after studying the first list, are told either to keep remembering the first list for a subsequent retention test or to forget the first list under the pretense that it was for practice only. At the time of the test, participants are informed that they should try to recall as many items as possible from both lists, irrespective of the original cuing. Typically, the forget cue leads to impaired memory of list 1 (pre-cue forgetting) and improved memory of list 2 (post-cue enhancement) relative to the remember cue (for reviews, see MacLeod, 1998; Bäuml et al., 2010; Sahakyan et al., 2013).

In recent years, the two effects of the forget cue have mostly been explained by retrieval inhibition or context change. The retrieval-inhibition account assumes that a cue to forget the previously learned list 1 triggers active inhibitory processes that impair access to the list 1 items and, as a result of reduced interference effects from these items, lead to improved memory of list 2 (Geiselman et al., 1983). Alternatively, proponents of the context-change account argue that the forget cue impedes list 1 recall because such cuing alters the subject's internal context and thus induces a mismatch between the list 1 context at encoding and the later test, and improves later list 2 recall because of the resulting interference release (Sahakyan and Kelley, 2002). More current two-mechanism accounts of LMDF—which attribute the two effects of the forget instruction to distinct underlying processes—also assume that list 1 forgetting is due to either retrieval inhibition or context change (see Sahakyan et al., 2013; Pastötter et al., 2017).

In the standard LMDF task, all list 1 items are designated as unimportant in the forget condition, and subjects are therefore asked to forget all pre-cue items. Previous studies that do so in fact reported forgetting of all pre-cue items, with similar levels of forgetting across the single list items (Sahakyan and Foster, 2009; Pastötter and Bäuml, 2010; Pastötter et al., 2012). Employing a variant of the standard LMDF task, however, more recent work examined whether such pre-cue forgetting could also be selective. That is, when participants have been presented with both relevant and irrelevant pre-cue information, are they able to forget the irrelevant pre-cue information while keeping in mind the relevant pre-cue information?

Selectivity of LMDF is theoretically important because different predictions can arise from different LMDF accounts. For instance, the context-change account predicts that selective LMDF should not be possible, because in response to the forget cue, an encoding-retrieval mismatch for all pre-cue items should arise, regardless of whether the items are all to be forgotten or consist of a mixture of relevant and irrelevant information. The retrieval-inhibition account, in itself, makes no unequivocal prediction on whether or not LMDF should be selective. Prior studies demonstrating that performance in the LMDF task can be related to individuals' working memory capacity (Delaney and Sahakyan, 2007; Soriano and Bajo, 2007; Aslan et al., 2010) and executive control function (Conway et al., 2000; Conway and Fthenaki, 2003; Hanslmayr et al., 2012) indicate, however, that retrieval inhibition constitutes a relatively flexible executive control mechanism that may be targeted in a selective way at the irrelevant pre-cue information. If so, participants may show selective LMDF.

Research on selective LMDF examined selectivity in two experimental tasks, the 2-list task and the 3-list task. In the 2-list task, subjects are shown relevant and irrelevant items in an alternating manner within a single list (list 1) and, after presentation of that list, are told to forget the irrelevant items but keep remembering the relevant ones. Afterwards, a second list consisting of relevant items only is presented. Delaney et al. (2009) examined selectivity by employing short three-word sentences and demonstrated that while forgetting of irrelevant list 1 items occurred, memory of relevant list 1 items remained intact, a finding that challenges the context-change account. Four more recent studies have provided additional evidence against the context-change account, by replicating this pattern of results using material similar to as well as different from that employed by Delaney et al. (Gómez-Ariza et al., 2013; Kliegl et al., 2013; Aguirre et al., 2014, 2017). In contrast, using material the same as and different from that of Delaney et al., two studies (Storm et al., 2013; Akan and Sahakyan, 2018) failed to detect any evidence of selectivity in the task, and found neither forgetting of relevant nor forgetting of irrelevant pre-cue information.

In the 3-list task of selective LMDF, subjects study three lists of items, with list 1 consisting only of relevant information and list 2 consisting only of irrelevant information. After presentation of list 2, participants are cued to forget the irrelevant list 2 but keep remembering the relevant list 1. Subsequently, they are presented with a third list that contains only relevant items. In this type of task, Sahakyan (2004) presented participants with lists of 12 items each. After presentation of each of the three lists, subjects received a cue to either forget or keep remembering the immediately preceding list. In the remember-remember-remember (RRR) condition participants were cued to remember each single list, whereas in the remember-forget-remember (RFR) condition they were cued to remember list 1 and list 3 but to forget list 2. The results showed non-selective forgetting of both list 1 and list 2 in the RFR condition, which is in better agreement with the context-change than with the retrieval-inhibition account. In contrast, more recent work reported evidence for selectivity in this task when relatively short pre-cue lists of six unrelated items each were employed (Kliegl et al., 2013). Again, there was an RRR condition, in which participants were cued to remember both list 1 and list 2, and an RFR condition, in which they were cued to forget list 2 but keep in mind list 1. Across three experiments, the results consistently showed evidence for selective LMDF: forgetting of list 2, but not of list 1, arose in the RFR condition, which is in better agreement with the retrieval-inhibition than with the context-change account. The pattern that emerged was independent of the modality in which the three lists had been presented in the study phase, and independent of whether the items of list 1 and list 2 had been presented in the same font color or different font colors and whether they had been read out loud by the same or different speakers.

The results from previous studies are thus mixed and do not provide a simple yes/no answer as to whether LMDF is selective or not. Rather, they may indicate that selectivity is present under some circumstances but absent under others. Although to date it is far from clear exactly which factors induce selective LMDF and which induce non-selective LMDF, the previous work provides us with some first clues to the question. For instance, Kliegl et al. (2013) kept the material and other procedural details constant and found equivalent selectivity in the 2-list and 3-list tasks of LMDF, indicating that the type of task may not have an influence on selectivity. An analogous indication arises for material, because selectivity in the 2-list task was reported for both sentences (Delaney et al., 2009; Aguirre et al., 2020) and simple word lists (Kliegl et al., 2013).

However, the results from prior work also suggest a factor that may influence selectivity in the task, namely the length of pre-cue lists. Sahakyan (2004) used longer pre-cue lists and found non-selective LMDF, whereas Kliegl et al. (2013) employed relatively short pre-cue lists and found selective LMDF, suggesting that shorter pre-cue lists may improve discriminability of lists and thus improve selectivity in LMDF (see Kliegl et al., 2013, p. 461). Because the studies by Sahakyan and Kliegl et al. have a number of methodological differences, concluding from the previous results that pre-cue list length can modulate selectivity would be premature, however. The primary aim of the present study is therefore to address the issue directly and examine whether length of pre-cue lists can influence selectivity in LMDF (see Experiment 2 below). The first goal of the present study is to provide a conceptual replication of the Kliegl et al. finding that selectivity in the 3-list LMDF task can arise with short pre-cue lists, using different word material and a different mode of item presentation than that earlier study (see Experiment 1 below). Such an attempt seems worthwhile given the importance of reproducibility in psychological studies (e.g., Johnson et al., 2017; Spellman and Kahneman, 2018).

## 2. Experiment 1

Following Experiment 2 of the Kliegl et al. (2013) study, this experiment examines selective LMDF by comparing the effects of three cuing conditions. Subjects were asked to study three lists consisting of unrelated words and, following study of list 2, were told to either keep remembering both list 1 and list 2 (RRR condition), forget both list 1 and list 2 (FFR condition), or forget list 2 but keep remembering list 1 (RFR condition). The RRR and FFR conditions serve as upper and lower baselines, allowing us to determine minimum (RRR) and maximum (FFR) forgetting of relevant and irrelevant pre-cue information in the RFR condition. On the basis of prior LMDF work (e.g., Geiselman et al., 1983) and Experiment 2 of the Kliegl et al. (2013) study, we expected that, relative to the RRR condition, (i) memory of both pre-cue lists would be impaired in the FFR condition—thus reflecting standard LMDF—and, more important, (ii) memory of the second pre-cue list would be impaired but memory of the first pre-cue list would be unaffected in the RFR condition—reflecting selectiv LMDF.

### 2.1. Method

#### 2.1.1. Participants

Following Experiment 2 of the Kliegl et al. (2013) study, we tested 30 participants in each of the three experimental conditions (RRR, RFR, and FFR). The 90 subjects (mean age 25.0 years, standard deviation 7.3 years, 62 females) were recruited from Regensburg University. All participants were tested individually.

#### 2.1.2. Material

As in Kliegl's et al. (2013) Experiment 2, 24 unrelated German nouns of medium frequency were drawn from the CELEX database, using the Wordgen v1.0 software toolbox (Duyck et al., 2004). Different nouns were used than in the previous study. For each participant, three item lists were prepared, with lists 1 and 2 consisting of six items each and list 3 consisting of 12 items. For all participants, the assignment of items to lists was random. The study material can be downloaded at https://osf.io/em75n/.

#### 2.1.3. Design

The experiment had a single-factor design with the between-subjects variable of cuing condition (RRR, RFR, FFR). In the RRR condition, list 2 was followed by a cue to remember both list 2 and list 1; in the RFR condition, list 2 was followed by a cue to forget list 2 but remember list 1; in the FFR condition, list 2 was followed by a cue to forget both list 2 and list 1.

#### 2.1.4. Procedure

The multiple-cue version of LMDF was used (see Zellner and Bäuml, 2006; Pastötter and Bäuml, 2007). Participants were told that they would be presented with lists of words to learn for a later recall test and that following the presentation of each list, they would be given a cue to remember or forget previously studied information. Further, participants were informed that a to-be-forgotten list would not be tested on the later recall test. At the start of the experiment, the experimenter sat in front of the participant and read out loud the items of the three lists with a presentation rate of 4 s per item. Prior to the presentation of each single list, participants were told: “I am now going to read to you list x. Please try to remember the words on the list as well as possible.” Item order within lists was random for all participants. Immediately after list 1 encoding, the experimenter asked the participant to keep remembering the list. After list 2 encoding, participants were told either to remember list 2 and keep on remembering list 1 (RRR), to forget list 2 but keep on remembering list 1 (RFR), or to forget both list 2 and list 1 (FFR). Following the encoding phase, participants counted backward from a three-digit number in steps of threes for 30 s as a recency control. At test, participants were asked to recall the three lists' items, irrespective of original cuing. To control the output order of pre-cue item lists, half of the participants were asked to recall list 1 items first and list 2 items second, and for the other half list output order was reversed. Because the focus of this study is on pre-cue item recall, participants were asked to recall pre-cue lists first. All participants were asked to recall list 3 items last. Participants wrote down the items of the three lists on separate unlabeled sheets of paper. Recall time for both list 1 and list 2 items was 30 s each, whereas recall time was 60 s for list 3. Procedural details of the experiment were identical to those in Experiment 2 of the Kliegl et al. (2013) study, the only major difference being that in the present experiment, the single word lists were read aloud “live” by the experimenter, whereas in the earlier experiment participants listened to the study lists from prerecorded audio files.

### 2.2. Results

**Figure 1:**
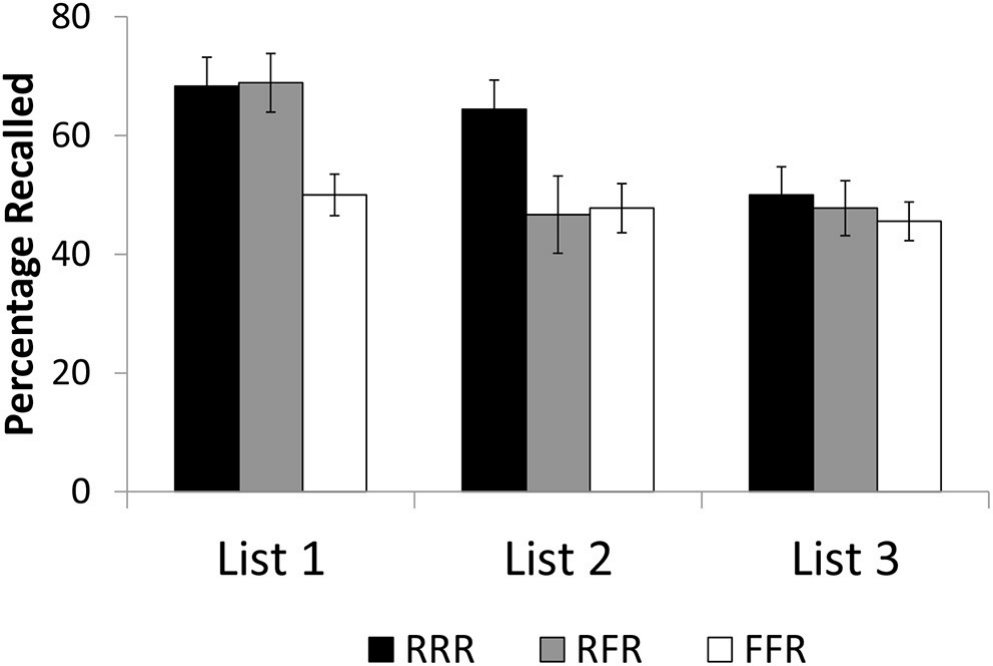
Mean recall rates as a function of cuing (RRR, RFR, FFR) in Experiment 1, separately for the three item lists. RRR = participants were asked to remember all three item lists; RFR = participants were asked to remember list 1 and list 3 but to forget list 2; FFR = participants were asked to forget list 1 and list 2 but to remember list 3. Error bars represent standard errors of the mean.

Figure 1 shows mean recall rates as a function of cuing (RRR, RFR, FFR), separately for the three lists. Items were counted as recalled if recalled with the correct list. All data can be downloaded at https://osf.io/em75n/.

#### 2.2.1. List 1 Recall

An overall ANOVA of the three cuing conditions (RRR, RFR, FFR) showed a main effect of cuing, *F*_(2,87)_ = 5.478, MSE = 0.063, *p* = 0.006, η^2^ = 0.112. Pairwise comparisons showed that list 1 recall rates in the RRR condition (68.3%) were higher than in the FFR condition (50.0%), *t*_58_ = 3.003, *p* = 0.004, *d* = 0.775. In addition, recall of list 1 items in the RFR condition (68.9%) was similar to that in the RRR condition (68.3%), *t*_58_ < 1, but higher than in the FFR condition, *t*_58_ = 3.058, *p* = 0.003, *d* = 0.800. As in Experiment 2 of the Kliegl et al. (2013) study, these results indicate that list 1 forgetting was present in the FFR condition but absent in the RFR condition.

#### 2.2.2. List 2 Recall

An overall ANOVA of the three cuing conditions (RRR, RFR, FFR) showed a significant main effect of cuing, *F*_(2,87)_ = 3.331, MSE = 0.089, *p* = 0.040, η^2^ = 0.071. Pairwise comparisons showed that list 2 recall rates in the RRR condition (64.4%) were higher than in the FFR condition (47.8%), *t*_58_ = 2.578, *p* = 0.013, *d* = 0.665, and in the RFR condition (46.7%), *t*_58_ = 2.092, *p* = 0.041, *d* = 0.540. List 2 recall did not differ between the FFR and RFR conditions, *t*_58_ < 1. The findings again replicate Experiment 2 of Kliegl et al. (2013), in showing that list 2 forgetting was present in both the FFR condition and the RFR condition.

#### 2.2.3. List 3 Recall

An overall ANOVA of the three cuing conditions (RRR, RFR, FFR) showed no main effect of cuing, *F*_(2,87)_ < 1, again replicating Experiment 2 of Kliegl et al. (2013).

#### 2.2.4. Intrusions

Table 1 shows intrusion rates in Experiment 1, separately for the three item lists. A list's intrusion rate is the percentage of study items that were erroneously recalled with the list. The list 1 intrusion rate, for example, refers to the number of items from lists 2 and 3 that were falsely recalled during the test of list 1, relative to the number of list 1 items that were presented. Three overall ANOVAs with the factor of cuing (RRR vs. RFR vs. FFR) showed no main effects for lists 1, 2, and 3, all with *F* ≤ 2.015. Again, intrusion rates were generally low, on the order of 4% in the single conditions, independent of cuing.

**Table 1:** Mean intrusion rates (and standard errors) as a function of cuing and length in Experiments 1 and 2.

		**List 1**		**List 2**		**List 3**	
		**Short**	**Long**	**Short**	**Long**	**Short**	**Long**
Experiment 1	RRR	3.3 (1.5)		1.7 (1.2)		2.2 (1.1)	
	RFR	3.3 (1.9)		6.7 (2.3)		3.1 (1.5)	
	FFR	5.0 (1.8)		3.9 (1.5)		1.7 (0.7)	
Experiment 2	RRR	3.6 (0.9)	4.0 (0.9)	3.6 (1.2)	1.8 (0.6)	2.2 (0.8)	4.6 (1.4)
	RFR	4.2 (1.2)	4.0 (1.0)	3.6 (1.2)	5.4 (1.3)	1.5 (0.5)	1.8 (0.6)
	FFR	4.5 (1.2)	4.2 (1.0)	4.8 (1.2)	3.3 (0.9)	4.5 (1.1)	3.1 (1.1)

### 2.3. Discussion

Consistent with our expectations, the results of Experiment 1 showed typical forgetting of irrelevant pre-cue items. Indeed, when participants were asked to forget both pre-cue lists in the FFR condition, later recall of both list 1 and list 2 was impaired; when participants were asked to forget list 2 in the RFR condition, later recall of list 2 was impaired, whereas later recall of list 1 remained intact. The results for the RFR condition thus replicate the Kliegl et al. (2013) findings, again demonstrating selective LMDF in this condition, i.e., decreased retention of irrelevant pre-cue items and intact retention of relevant pre-cue items. The results of Experiment 1 challenge the context-change account, according to which forgetting of all pre-cue information should occur. The context change after list 2 should cause a mismatch between pre-cue encoding context and the context at test, which should reduce recall of both list 1 and list 2. In contrast, the results can be reconciled with the retrieval-inhibition account, at least under the assumption that inhibition is triggered by a flexible executive-control mechanism, as may be indicated by the results of previous studies (e.g., Aslan et al., 2010; Hanslmayr et al., 2012). If so, forgetting of the irrelevant pre-cue items but not the relevant pre-cue items may occur.

Our failure to find enhancement of the list 3 post-cue items aligns with the findings of several recent LMDF studies (e.g., Zellner and Bäuml, 2006; Delaney and Sahakyan, 2007) and prior selective LMDF studies (e.g., (Delaney et al., 2009; Kliegl et al., 2013), Experiments 2 and 3). Typically, failure to observe post-cue enhancement in LMDF arises when—as in the current experiment—the pre-cue items are recalled prior to the post-cue items. In fact, Pastötter et al. (2012) conducted a meta-analysis on the role of list output order in LMDF which found that the forget cue improves post-cue item recall mainly when the post-cue items are tested first and shows hardly any enhancement effect when the post-cue items are tested last. The absence of an enhancement effect in the present experiment was therefore probably a consequence of the chosen list output order.

## 3. Experiment 2

Experiment 2 examines whether selectivity in the 3-list LMDF task is affected by length of pre-cue lists. As in Experiment 1, we employed the RRR, FFR, and RFR conditions. Again, the RRR and FFR conditions served as minimum and maximum forgetting baselines, against which recall of the relevant and irrelevant pre-cue items in the RFR and FRR conditions were compared. In the 6-6-12 condition, participants were exposed to relatively short pre-cue lists, consisting of 6 items each, thus replicating the conditions of the Kliegl et al. (2013) study and the present Experiment 1. In contrast, in the 12-12-12 condition, participants were presented with longer pre-cue lists, consisting of 12 items each, thus mimicking the conditions in the Sahakyan (2004) study. List 3 consisted of 12 items in both conditions. Following Sahakyan (2004, Experiment 1), Kliegl et al. (2013, Experiments 1–3), and the present Experiment 1, list output order was controlled at test and participants were asked to recall the pre-cue items first and the post-cue items last.

Based on the findings of Kliegl et al. (2013) and our Experiment 1, we expected that participants would show selective LMDF in the 6-6-12 condition. If so, cuing them to selectively forget list 2 should induce forgetting of the irrelevant pre-cue list (list 2) but not induce forgetting of the relevant pre-cue list (list 1). On the basis of the Sahakyan (2004) finding and the hypothesis that pre-cue list length can affect selectivity in LMDF, we expected selectivity to be reduced, or even absent, in the 12-12-12 condition. The results of the experiment will provide direct evidence on the possible effect of pre-cue list length on selectivity in LMDF.

### 3.1. Method

#### 3.1.1. Participants

The participants were 336 students at Regensburg University (mean age 28.3 years, standard deviation 11.5 years, 201 females). They were tested individually, with 56 participants in each of the six experimental conditions. Indeed, an analysis of test power conducted with the GPower program (version 3; Faul et al., 2007) revealed that to detect at least a small to medium-sized effect (*f* = 0.20; Cohen, 1988) for the critical interaction with a probability of 1−β = 0.90 and α = 0.05, 54 participants are required in each group.

#### 3.1.2. Material

The same 24 unrelated German nouns as in Experiment 1 were used, and an additional 12 unrelated German nouns were drawn from the CELEX database using the Wordgen v1.0 software toolbox (Duyck et al., 2004). Three lists of 12 words each (list A, list B, and list C) were created. List A and list B were further split into two sublists of six words each (sublists A1, A2, B1, and B2). Across lists and sublists, words were matched in frequency and length. In contrast to Experiment 1, assignment of items to lists and sublists was constant for all participants. Lists A and B, and each of the four sublists, served exclusively and equally often as lists 1 and 2. List C always served as list 3. Each list was used equally often in the RRR condition, the RFR condition, and the FFR condition. The study material can be downloaded at https://osf.io/em75n/.

#### 3.1.3. Design

The experiment had a 3 × 2 design with the between-subjects factors of cuing (RRR, RFR, FFR) and list length (6-6-12, 12-12-12). In the RRR condition, list 2 was followed by a cue to remember both list 2 and list 1; in the RFR condition, list 2 was followed by a cue to forget list 2 but remember list 1; in the FFR condition, list 2 was followed by a cue to forget both list 2 and list 1. Regarding list length, conditions differed in the number of list 1 and list 2 items: in the 6-6-12 condition, list 1 and list 2 consisted of 6 items each; in the 12-12-12 condition, list 1 and list 2 consisted of 12 items each. List 3 always consisted of 12 items.

#### 3.1.4. Procedure

The procedure was identical to that in Experiment 1, the only differences being that at study, half of the participants were presented with short pre-cue lists (6-6-12 condition) and the other half with long pre-cue lists (12-12-12 condition), and at test, recall time for both list 1 and list 2 items was 30 s each in the 6-6-12 condition and 60 s in the 12-12-12 condition.

### 3.2. Results

**Figure 2:**
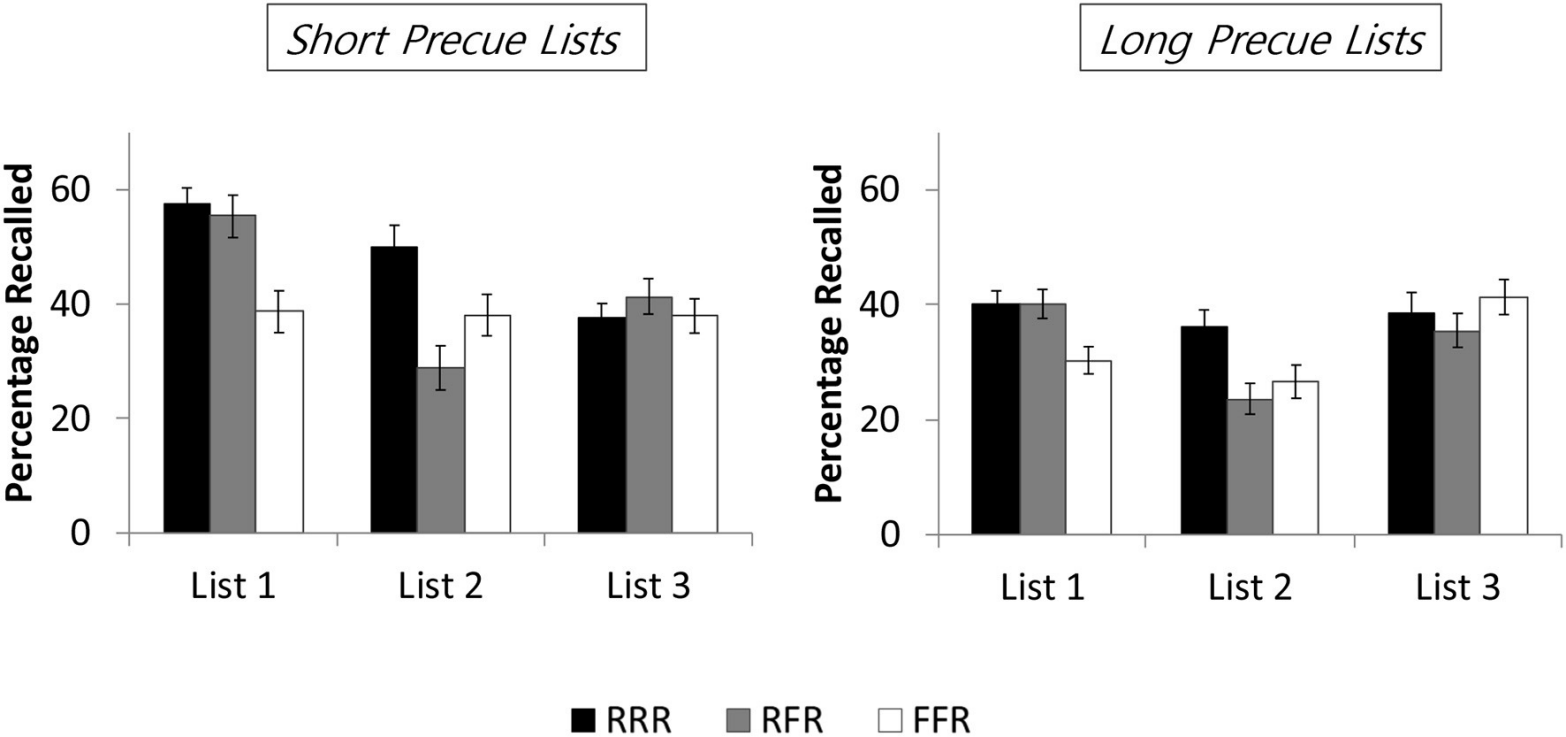
Mean recall rates as a function of cuing (RRR, RFR, FFR) and pre-cue list length (short, long) in Experiment 2, separately for the three item lists. RRR = participants were asked to remember all three item lists; RFR = participants were asked to remember list 1 and list 3 but to forget list 2; FFR = participants were asked to forget list 1 and list 2 but to remember list 3. Error bars represent standard errors of the mean.

Figure 2 shows mean recall rates as a function of cuing (RRR, RFR, FFR) and list length (6-6-12, 12-12-12), separately for the three lists. Items were counted as recalled if recalled with the correct list.

#### 3.2.1. List 1 Recall

A 3 × 2 ANOVA with the factors of cuing (RRR vs. RFR vs. FFR) and list length (6-6-12 vs. 12-12-12) revealed a main effect of cuing, *F*_(2, 330)_ = 14.449, MSE = 0.049, *p* < 0.001, partial η^2^ = 0.081, and a main effect of list length, *F*_(1, 330)_ = 31.560, MSE = 0.049, *p* < 0.001, partial η^2^ = 0.087, but no interaction between the two factors, *F*_(2, 330)_ = 1.243, MSE = 0.049, *p* = 0.290, partial η^2^ = 0.007. List 1 recall rates for the short lists were higher than for the long lists (59.5 vs. 36.9%), *t*_334_ = 5.40, *p* < 0.001, *d* = 0.59. Regarding the main effect of cuing, pairwise comparisons showed that list 1 recall rates in the FFR condition (34.5%) were lower than in both the RRR condition (48.8%), *t*_222_ = 4.791, *p* < 0.001, *d* = 0.640, and the RFR condition (47.8%), *t*_222_ = 4.116, *p* < 0.001, *d* = 0.550; list 1 recall rates did not differ between the RFR and RRR conditions, *t*_222_ < 1. These results indicate that list 1 forgetting was present in the FFR condition but absent in the RFR condition, for both the short and the long pre-cue lists.

#### 3.2.2. List 2 Recall

A 3 × 2 ANOVA with the factors of cuing (RRR vs. RFR vs. FFR) and list length (6-6-12 vs. 12-12-12) showed a main effect of cuing, *F*_(2, 330)_ = 12.983, MSE = 0.063, *p* < 0.001, partial η^2^ = 0.073, and a main effect of list length, *F*_(1, 330)_ = 13.894, MSE = 0.063, *p* < 0.001, partial η^2^ = 0.040, but no interaction, *F*_(2, 330)_ < 1. List 2 recall rates for the short lists were higher than for the long lists (40.0 vs. 28.8%), *t*_334_ = 3.60, *p* < 0.001, *d* = 0.39. Regarding the main effect of cuing, pairwise comparisons showed that compared to the RRR condition (43.1%), list 2 recall rates were lower in both the FFR condition (32.4%), *t*_222_ = 3.114, *p* = 0.002, *d* = 0.416, and the RFR condition (26.3%), *t*_222_ = 4.905, *p* < 0.001, *d* = 0.656; list 2 recall did not differ reliably between the FFR and RFR conditions, *t*_222_ = 1.825, *p* = 0.069, *d* = 0.244. These results indicate that list 2 forgetting was present in both the FFR and the RFR conditions, for both the short and the long pre-cue lists^1^.

#### 3.2.3. List 3 Recall

A 3 × 2 ANOVA with the factors of cuing (RRR vs. RFR vs. FFR) and list length (6-6-12 vs. 12-12-12) showed no main effect of cuing, *F*_(2, 330)_ < 1, no main effect of list length, *F*_(1, 330)_ < 1, and no interaction between the factors, *F*_(2, 330)_ = 1.22, MSE = 0.05, *p* = 0.30, partial η^2^ = 0.01. Neither list length of the pre-cue lists nor cuing affected list 3 recall.

#### 3.2.4. Intrusions

Table 1 shows intrusion rates, separately for the three item lists. Three 3 × 2 ANOVAs with the factors of cuing (RRR vs. RFR vs. FFR) and list length (6-6-12 vs. 12-12-12) showed no main effects or interactions, for all three item lists, with all *F* ≤ 2.79. Intrusion rates were generally low, on the order of 4% in the single conditions, independent of cuing and list length.

### 3.3. Discussion

The results of Experiment 2 show typical directed forgetting of the irrelevant pre-cue items. In the FFR condition, cuing participants to forget the two pre-cue lists induced forgetting of both list 1 and list 2; in the RFR condition, cuing participants to forget list 2 induced forgetting of list 2. These effects were similarly present for both short and long pre-cue lists. More important, in the RFR condition, forgetting of (relevant) list 1 was absent for both short and long pre-cue lists, demonstrating that selective forgetting occurred and that it did not depend on pre-cue list length. The results for the short pre-cue lists replicate the findings of Kliegl et al. (2013) and the present Experiment 1. The results for the long pre-cue lists extend the prior work, indicating that pre-cue list length does not play a critical role in selectivity. The finding of selectivity for long pre-cue lists, of course, contrasts with Sahakyan's (2004) original failure to find selectivity for long lists, suggesting that other procedural differences between the studies may have caused the discrepancy in results (see General Discussion). Similar to the results of Experiment 1, the results of Experiment 2 seem difficult to reconcile with the context-change account, but may be consistent with the retrieval-inhibition account.

## 4. General Discussion

Prior work examining selectivity in LMDF yielded mixed results. Whereas, some studies found evidence of selectivity (Delaney et al., 2009; Gómez-Ariza et al., 2013; Kliegl et al., 2013, 2018; Aguirre et al., 2014, 2017), in other studies no selectivity was reported (Sahakyan, 2004; Storm et al., 2013; Akan and Sahakyan, 2018). Moreover, one of the studies that reported non-selectivity found forgetting of both relevant and irrelevant pre-cue information (Sahakyan, 2004), whereas two other studies reported neither forgetting of relevant nor forgetting of irrelevant pre-cue information (Storm et al., 2013; Akan and Sahakyan, 2018). Although, as a whole, these findings indicate that under certain circumstances LMDF can be selective, to date it is largely unclear which factors play a critical role in selectivity and which factors do not.

Against this background, the present study directly examines the role of one critical factor that may influence selectivity in LMDF, namely length of pre-cue lists. We hypothesized that selectivity could benefit from short pre-cue lists and thus be present with short lists but absent with long lists. This hypothesis was motivated by the results of previous studies which, in the 3-list task, found no selectivity for longer pre-cue lists (Sahakyan, 2004) but did find selectivity for short pre-cue lists (Kliegl et al., 2013). Using the same pre-cue list lengths as in the two previous studies, however, the present study did not find any evidence for an effect of pre-cue list length on selectivity in the 3-list task but rather demonstrated selectivity regardless of list length. The results of the present Experiments 1 and 2 replicate the results of Kliegl et al. (2013) for short pre-cue lists and extend them to longer pre-cue lists. At the same time, the results disagree with those of Sahakyan (2004), suggesting that factors other than pre-cue list length may be responsible for the discrepancy.

One such factor may be control of list output order. While in the present study the pre-cue lists were always recalled first and the post-cue list last, participants in Sahakyan's (2004) Experiment 2, for instance, were allowed to recall the three lists' items in any order they wished. Doing so, subjects in the remember condition (RRR) may have tended to recall list 1 items first, whereas subjects in the selective forget condition (RFR) may have tended to recall the post-cue items first (e.g., Geiselman et al., 1983). If so, output interference may have contributed to list 1 recall in the remember condition but not in the selective forget condition and been responsible for the failure to find selectivity in the task. However, differences in output interference cannot explain all the inconsistency between studies, because in Experiment 1 Sahakyan (2004) did control for output interference but did not find evidence for selectivity. Since comparisons between studies are generally difficult, future work should investigate the effect of output order of pre-cue and post-cue lists on selective LMDF within a single experiment.

The results from recent work on selectivity in LMDF suggest two factors that may leave the degree of selectivity largely unaffected. One factor is the type of LMDF task and the other factor is material. Indeed, comparing selectivity in the 2-list and 3-list tasks directly, Kliegl et al. (2013) found equivalent degrees of selectivity, indicating that results from the 2-list task may generalize to the 3-list task, and vice versa. Similarly, some previous studies reporting selectivity in the 2-list task used unrelated items whereas others used short sentences (Delaney et al., 2009; Kliegl et al., 2013), indicating that material may not play a critical role in selectivity. By demonstrating selectivity for both short and long pre-cue lists, the present study adds amount of pre-cue information to the list of factors that do not seem to influence selectivity in LMDF.

The present results are not easily explained by the context-change account, which claims that the forget cue induces a change in the subject's mental context and thus impairs recall of the pre-cue items because of a mismatch between the context at encoding and the context at test (Sahakyan and Kelley, 2002). Therefore, no selectivity should arise and both the irrelevant and the relevant pre-cue information would be subject to forgetting. The present results turned out otherwise, however, demonstrating selective forgetting of irrelevant pre-cue information without affecting memory of the relevant pre-cue information. The retrieval-inhibition account suggests that forget-cued participants engage in active inhibitory processes that reduce access to list 1 items (Geiselman et al., 1983). If inhibition reflects the action of a relatively flexible control mechanism (Aslan et al., 2010; Hanslmayr et al., 2012), one might expect that retrieval inhibition would induce selective forgetting of irrelevant pre-cue information, which is what the results of the present experiments show.

Recent work on the effects of prolonged retention interval on standard LMDF reported evidence that list 1 forgetting is not short-lived and is still present after delays of 20 min or even 24 h (Abel and Bäuml, 2017, 2019). Like the present results, this finding challenges the context-change account, because mental context change is generally assumed to produce relatively transient forgetting (e.g., Sahakyan and Kelley, 2002; Divis and Benjamin, 2014). Whatever the exact mechanisms may be that underlie this forgetting (for a discussion, see Abel and Bäuml, 2019), if standard LMDF and selective LMDF were mediated by the same mechanisms, then not only standard LMDF but also selective LMDF should be lasting, and the present results would thus generalize to delays of at least 20 min. Persistence of selective LMDF has not been investigated to date and future work is therefore required to examine this critical prediction.

*In sum*, the present findings indicate that selective LMDF in the 3-list task arises not only for short lists but also for relatively long lists. These findings confirm and extend prior work on selectivity, which showed robust selective LMDF with short lists in both the 3-list task (Kliegl et al., 2013) and the 2-list task (Delaney et al., 2009; Gómez-Ariza et al., 2013; Kliegl et al., 2013; Aguirre et al., 2020). Overall, the findings suggest that, in many situations, people can flexibly forget a fraction of previously studied material without affecting memory of the remaining material. Theoretically, this pattern of results is difficult to reconcile with the context-change account, while being basically consistent with a retrieval-inhibition view of LMDF.

## Data Availability Statement

All data can be found at Open Science Framework, https://osf.io/em75n/.

## Ethics Statement

Ethical review and approval was not required for the study on human participants in accordance with the local legislation and institutional requirements. Written informed consent for participation was not required for this study in accordance with the national legislation and the institutional requirements.

## Author Contributions

OK, BP, and K-HB developed the study concept and experimental design. OK organized the data collection, performed the data analysis, and drafted the manuscript. BP and K-HB gave critical input for various revisions of the manuscript. All authors approved the final version of the manuscript for submission.

## Conflict of Interest

The authors declare that the research was conducted in the absence of any commercial or financial relationships that could be construed as a potential conflict of interest.

## Footnotes

^1^Visual inspection of Figure 2 seems to indicate that the amount of forgetting of list 2 items in the RFR condition, in comparison to the RRR condition, is more pronounced for the short pre-cue lists than the long pre-cue lists (list 2 forgetting: 21.1 vs. 12.5%). However, a 2 × 2 ANOVA with the factors of cuing (RRR vs. RFR) and list length (6-6-12 vs. 12-12-12) showed no interaction between the two factors, *F*_(1, 220)_ = 1.639, MSE = 0.064, *p* = 0.202, η^2^ = 0.007, suggesting that the RFR cue did not induce significantly greater forgetting for the short than for the long list 2. Also note that when converted to the number of items forgotten, participants forgot about 1.27 list 2 items in the RFR condition relative to the RRR condition when short lists were used, and 1.50 list 2 items in the FFR condition relative to the RRR condition when long lists were used. Thus, regardless of how forgetting is coded, the amount of list 2 forgetting appears quite comparable for short and long pre-cue lists.
